# Modulation of Neural Activity for Myelination in the Central Nervous System

**DOI:** 10.3389/fnins.2019.00952

**Published:** 2019-09-06

**Authors:** Elliot H. Choi, Agata Blasiak, Joonho Lee, In Hong Yang

**Affiliations:** ^1^Department of Pharmacology, School of Medicine, Case Western Reserve University, Cleveland, OH, United States; ^2^Department of Ophthalmology, School of Medicine, Gavin Herbert Eye Institute, University of California, Irvine, Irvine, CA, United States; ^3^Department of Biomedical Engineering, National University of Singapore, Singapore, Singapore; ^4^School of Medicine and Dentistry, University of Rochester, Rochester, NY, United States; ^5^Department of Mechanical Engineering and Engineering Science, Center for Biomedical Engineering and Science, University of North Carolina at Charlotte, Charlotte, NC, United States

**Keywords:** electrical stimulation, optogenetic stimulation, magnetic stimulation, myelination, neural activity, oligodendrocyte, Schwann cells

## Abstract

Electrical stimulation has been playing a significant role in revealing various functions and mechanisms of the nervous system. It is no different for myelination, a process in which oligodendrocytes in the central nervous system (CNS) or Schwann Cells in the peripheral nerve system (PNS) wrap around axons to provide an insulating layer *in vitro* and *in vivo*. It has been widely recognized that the myelin sheath accelerates axon signal conduction and provides neuroprotection. Recent studies have begun to reveal its role in plasticity. The major mechanism that enables this process is activity-dependent myelination – the phenomenon where neuronal activity supports oligodendrocyte maturation and myelin sheath formation. In light of recent discoveries, a better understanding of this phenomenon has a potential to provide therapeutic targets for not only demyelinating diseases, but also psychiatric disorders. There is a growing need for experimental platforms capable of dissecting the effect of neural activity on myelination in health and disease. The effect of neural activity is commonly studied by comparing the myelination levels in cultures with neurons of low and high activity. Electrical stimulation is particularly well suited as a method of inducing neural activity in these systems. In this review, we describe *in vitro* platforms for studying activity-dependent myelination, which utilize neuron stimulation via electrical field. We also discuss stimulation profiles, as well as the alternatives to electrical stimulation in the context of regular, compartmentalized, and organotypic co-cultures.

## Introduction

Neuromodulation is an emerging technique for treating neurological diseases and psychiatric disorders in the field of medicine. This technique was first introduced by Merton and Morton (1980) who employed a high-voltage electrical stimulator to stimulate the primary cortex in humans through the intact scalp. Upon stimulation, a weak muscle twitch on the contralateral hand was generated (Merton and Morton, 1980). It became apparent that the stimulation could activate muscle fibers by inducing upper motor neuron activities. This observation provided the evidence that non-invasive brain stimulation would be useful in both research and medicine. Over the past 30 years, numerous clinical studies have been performed with different techniques including transcranial electrical stimulation (TES) and transcranial magnetic stimulation (TMS). TES applies constant, oscillating or randomly alternating currents through two or more electrodes to modulate brain activity. The predominant direction of the current is radial to the brain surface. For TMS, stimulation is produced by a brief, high-intensity magnetic field, which is generated by an electric current passing through a magnetic coil. In contrast to TES, the predominant direction of the current is tangential to the brain surface. In recent years the application of neuromodulation has been extensively expanding as more studies demonstrate its therapeutic potential for treating a host of maladies, including major depressive disorders, obsessive-compulsive disorder, stroke, epilepsy, Parkinson’s disease, and Alzheimer’s disease (Tergau et al., 1999; George et al., 2010; Khedr et al., 2010; Mantovani et al., 2010; Rabey et al., 2013; Torres et al., 2015). The therapeutic effect mainly results from modulating neural activity. Low frequency stimulation reduces neural activity while high frequency stimulation excites neural activity (Hallett, 2007). It is clear that the effect of electrical stimulation is not limited to neurons. Multiple studies have suggested that electrical stimulation affects oligodendrocytes or Schwann cells which support neural conduction in the nervous system.

The nervous system of vertebrates requires rapid propagation of action potentials to integrate signals from the external environment. This rapid propagation is possible because of the myelination of axons, a process by which oligodendrocytes in the central nervous system (CNS) or Schwann cells in the peripheral nervous system wrap the axon. During the embryonic period, myelination begins with the proliferation of oligodendrocytes and Schwann cells followed by the establishment of glia-axon contacts (Jessen and Mirsky, 2005; Nave and Werner, 2014). Upon contact with the axonal membrane, OPCs and newly differentiated oligodendrocytes extend and retract their processes (Kirby et al., 2006). When contact is established, several molecular rearrangements result in a polarization of myelinating cells toward the axonal membrane (Baron and Hoekstra, 2010; Ozcelik et al., 2010; Nave and Werner, 2014). Longitudinal expansion of myelin segments coincides with secondary axon elongation during postnatal development (Hildebrand et al., 1993, 1994). After the peak of myelination in early life, remodeling of mature myelin membranes slows down. However, OPCs continue to proliferate and differentiate (Young et al., 2013) while Schwann cells retain high plasticity (Young et al., 2013; Jessen et al., 2015).

The mature myelin sheath is interrupted at regular intervals by unmyelinated regions where the membrane of the axon is exposed to the extracellular space. This arrangement of myelination allows for the generation of action potentials at short, unmyelinated axonal segments, and increases the velocity at which the action potentials are conducted. As such, small changes in myelin structure can lead to substantial changes in conduction velocity (Waxman and Bennett, 1972). In addition, myelin provides metabolic and trophic factors, which play a critical role in development of axons and viability of neurons (Wilkins et al., 2001, 2003; Funfschilling et al., 2012). Therefore, developmental failure of myelination in the nervous system in early life or loss of myelin have debilitating consequences in the remaining axons. It has been intriguing to examine whether modulating the activity of axons could induce myelination and hold therapeutic promise in demyelinating diseases. Interestingly, converging evidence has demonstrated that neural activity promotes oligodendrogenesis and myelination (Demerens et al., 1996; Stevens et al., 2002; Gibson et al., 2014; Mitew et al., 2018). Early support for this hypothesis emerged from the finding that transection of the developing optic nerve by axotomy or blockade of activity in the developing optic nerve by tetrodotoxin (TTX) dramatically reduces the rate of OPC proliferation (Barres and Raff, 1993) and the degree of optic nerve myelination (Demerens et al., 1996). In accordance with these studies, it was demonstrated that increasing neural activity with α-scorpion toxin enhances myelination (Demerens et al., 1996). Much of the *in vitro* work investigating the mechanisms by which neural activity regulates myelination has focused on the instructive roles of neurotransmitters and soluble factors. For instance, several studies have suggested that glutamate or acetylcholine released from depolarized neurons induces the synthesis of myelin (Gallo et al., 1996; Gudz et al., 2006; De Angelis et al., 2012). In dorsal root ganglion (DRG) neurons and OPC co-culture, adenosine was released from the neurons in an activity-dependent manner, promoting OPC differentiation, and myelination (Stevens et al., 2002). Moreover, brain-derived neurotrophic factor (BDNF) released from neurons has been shown to enhance myelin formation from oligodendrocytes and Schwann cells (Wan et al., 2010; Xiao et al., 2010). Building on these studies, development of new technologies has uncovered a rich experimental landscape for understanding neural activity-dependent myelination. Specifically, the compartmentalized microfluidic platform has become a valuable tool due to its applicability and flexibility (Campenot, 1977; Taylor et al., 2005; Wu et al., 2005; Park et al., 2006, 2014; Cox et al., 2008). The integration of co-cultures in the compartmentalized platform has enabled the physical separation of axons and oligodendrocytes from the neuronal soma (Yang et al., 2012; Malone et al., 2013; Lee et al., 2016; Prasad et al., 2017; Blasiak et al., 2018). In the present review, we discuss the role of neural activity in myelination and induction of myelination *in vitro* through stimulation of neurons with different technologies.

## Early Studies Elucidating the Role of Axons in Myelination

Oligodendrocytes in the CNS have the unique ability to form myelin. Although there is close interaction between oligodendrocytes and neurons, early evidence that neurons directly influence the formation of myelin was lacking. Several studies have suggested that cultured oligodendrocytes express proteins necessary for myelination and develop myelin-like structures even in the absence of neurons (Mirsky et al., 1980; Dubois-Dalcq et al., 1986). Moreover, oligodendrocytes isolated from rodent brain could extend their tips to form myelin-like structures (Sarlieve et al., 1980; Szuchet et al., 1986). However, contradictory lines of evidence called into question the validity of myelination in the absence of axons. Analysis of the myelin-like structures by electron microscopy demonstrated that these structures were not compactly organized compared to the myelin that wrap axons (Althaus et al., 1984; Lubetzki et al., 1993). Ultrastructural analysis showed that the processes of oligodendrocytes folded up on themselves rather than winding around themselves. Moreover, primary oligodendrocytes cultured with astrocytes and neurons specifically myelinated axons but not astrocyte processes or dendrites. This exclusive myelination of axons suggested that a molecular cue from axons may recruit oligodendrocyte processes (Lubetzki et al., 1993). Later, it became evident that the factors released from axons play a trophic role in the proliferation of oligodendrocyte progenitor cells (OPCs) and subsequent differentiation (Wood and Bunge, 1986; Lubetzki et al., 1992). Following axotomy of the optic nerve, oligodendrocytes clustered without their longitudinal orientation, developed fewer processes, and eventually failed to form myelin in the transected optic nerve (Ueda et al., 1999). These results suggested that viable axons are essential for three-dimensional organization of oligodendrocytes and myelination. Taken together, these pioneer reports strongly argued that axons play a role in myelination. However, conclusive evidence of whether neural activity could influence myelination remained elusive.

## Induction of Myelination by Neural Activity

Myelination is a finely orchestrated process that involves interactions of oligodendrocytes or Schwann cells with other cells through extracellular signaling and physical contacts. Thus, it would be logical to speculate that these myelinating cells synchronize their differentiation according to neural development and activity. In the 1960s, Gyllensten and Malmfors introduced the idea that neural activity could influence the function of oligodendrocytes. Their study demonstrated that mice reared in the dark developed fewer myelinated axons in the optic nerve compared with control mice (Gyllensten and Malmfors, 1963). Lack of myelination was also observed in the optic nerve of blind rats, whereas myelination was accelerated in the optic nerve by pre-mature eye opening (Tauber et al., 1980; Omlin, 1997). These findings were further supported by a similar study showing that blockade of action potentials with 10^–6^ M TTX inhibited myelination, whereas increase in duration and frequency of action potentials with 10^–9^ M α-scorpion toxin enhanced myelination (Demerens et al., 1996). The idea that neural activity can induce myelination has been further advanced by a number of recent studies that employed powerful genetic and imaging tools. For instance, channelrhodopsin 2 (ChR2) has been utilized as an optogenetic tool to manipulate neural activity since its discovery. ChR2 is a light-gated cation channel derived from photoreceptors in microalgae (Nagel et al., 2003). Because ChR2 can depolarize neurons within milliseconds with 470-nm light, expression of ChR2 in a specific group of neurons allows spatial and temporal regulation of neuronal activity (Boyden et al., 2005; Arenkiel et al., 2007). Optogenitic stimulation (cycles of 30 s on, 2 min off, 10 min/d for 7 days) of the premotor circuit in Thy1:ChR2 mice resulted in newly generated oligodendrocytes and increased thickness of the associated myelin sheath (Gibson et al., 2014). In accordance with this result, pharmacogenetic stimulation of somatosensory axons in the mouse brain almost doubled the number of mature oligodendrocytes capable of myelination (Mitew et al., 2018). Conversely, the study also demonstrated that attenuation of neural activity reduces myelination. Neural activity modulates myelination not only by directly stimulating oligodendrocytes but also by activating microglia and astrocytes (Ishibashi et al., 2006). Studies in both visual and auditory systems have demonstrated that neural activity induces the activation of microglia (Tremblay et al., 2010; Rosskothen-Kuhl et al., 2018). The activated microglia could promote myelination through clearance of the cellular debris that could potentially to interfere with myelination processes (Kotter et al., 2006; Church et al., 2017). In line with this, *Cx3cr1*^–/–^ mice exhibiting severe deficiency of microglia phagocytosis have impaired myelination (Lampron et al., 2015). Microglia also directly regulate proliferation, differentiation, and migration of OPCs (Miron, 2017). Taken together, recent studies strongly suggest that neural activity potentiates myelination. In line with the *in vivo* studies, several studies have elucidated potential molecular mechanisms mediating neural activity-dependent myelination. For instance, ATP released from DRGs in an activity-dependent manner is hydrolyzed to adenosine. Subsequently, adenosine binds to adenosine receptors on the OPC and promotes myelination (Stevens et al., 2002). There is also mounting evidence that neural activity triggers release of BDNF from axons and microglia (Trang et al., 2009; Parkhurst et al., 2013), which can subsequently induce myelination formation through the TrkB/Erk signaling pathway (Wan et al., 2010; Xiao et al., 2010; Ishii et al., 2012, 2013). Leukemia inhibitory factor (LIF) released from astrocytes in response to neural activity also appears to promote myelination (Ishibashi et al., 2006). Thus, it is clear that neural activity is an external regulator of myelination with important functional implications.

## Enhancement of Myelination Following Modulation of Neural Activity *in vitr*o

The therapeutic effect of TES and TMS has been attributed to its ability to modulate neural activity, which provides hope that TES and TMS can restore myelination via neural activity modulation. Although studies conducted over the past two decades collectively demonstrated that neural activity promotes myelination, a more complete understanding of activity-dependent myelination is essential for the development of activity-based therapies to treat demyelinating diseases. Moreover, there are very limited findings regarding the response of oligodendrocytes to TES or TMS. It is critical to carefully examine the influence of TES or TMS on oligodendrocytes, especially when they are simultaneously stimulated with neurons. To address the cellular and molecular mechanisms, *in vitro* models allowing electrical or optogenetic stimulation in neuron/oligodendrocyte co-culture have been developed (Ishibashi et al., 2006; Yang et al., 2012; Lee et al., 2016, 2017; Blasiak et al., 2018). These models benefit from a compartmentalized microfluidic platform which allows the isolation of neuronal cell bodies from axons and oligodendrocytes. The features of the compartmentalized microfluidic platform were leveraged for myelination studies to more accurately mimic the *in vivo* microenvironment, to stimulate neurons exclusively and to study the effect of a focal stimulation on different subcellular locations. When 10 Hz electrical stimulation was applied to DRGs for 7 days (1 h/day), the formation of myelin segments was increased by fivefold compared to the non-stimulated groups (Yang et al., 2012). The formation of myelin was also enhanced following electrical stimulation of DRGs (10 Hz, 1 h/day for 7 days) prior to introducing oligodendrocytes in the culture (Malone et al., 2013). This study also demonstrated 10 Hz to be the most effective stimulation frequency in the range of 1 to 100 Hz, and 7 days to be the most effective length of the stimulation course. These findings were consistent with previous studies showing an active role of neurons in myelination of their axons. The optimized stimulation parameters were also used to demonstrate that electrical stimulation enhances myelination independent of subcellular location (Lee et al., 2017). When electrical stimulation (10 Hz, 1 h/day for 3–14 days) was delivered to soma, proximal axons, or distal axons, the degree of myelination was similar regardless of the stimulation site, but higher than in non-stimulated neurons (Lee et al., 2017). Similarly, subcellular optogenetic stimulation was applied to study the effect of neural stimulation on myelination (Lee et al., 2016; Blasiak et al., 2018). In line with previous studies, focal stimulation (10 Hz, 1 h/day for 3–14 days) on neurons was sufficient to promote myelination of axons. Based on these findings, it is reasonable to speculate that neural stimulation of distal axons innervating muscles could be as effective as neural stimulation of soma in the spinal cord as a treatment for demyelinating diseases.

## Perspectives, Unanswered Questions and Concluding Remarks

In summary, we have provided an overview of the role of neural activity in myelination, with an emphasis on myelination via modulation of neural activity. The pioneering efforts in the field have unraveled the complex interactions between oligodendrocytes and neurons. Particularly, recent *in vivo* studies employing optogenetics and pharmacogenetics have provided strong evidence that stimulation of neural activity promotes myelination (Gibson et al., 2014; Mitew et al., 2018). Interestingly, stimulation of demyelinated axons could enhance oligodendrocyte differentiation and remyelination (Ortiz et al., 2019). While the role of neural activity in myelin formation has become apparent, many mechanistic details remain to be filled in through further investigations. Perhaps most important is the identification of factors involved in activity-dependent myelination, which will enable choosing targets for remyelination and lesion repair. Because stimulation of neural activity could enhance myelination in co-culture of neurons and OPCs, molecular mechanisms linking neural activity and myelin formation should be further studied with *in vitro* models.

As transcranial electrical stimulation and transcranial magnetic stimulation allow modulation of neuronal firing pattern, they could induce activity-dependent myelination. However, it is still unclear how the interaction between oligodendrocytes and neurons will be influenced when both cell types are simultaneously stimulated. It is conceivable that external stimuli might change the contents of soluble factors released from both oligodendrocytes and neurons, which in turn activate numerous signaling pathways. In addition, the external stimuli could abruptly change the cellular membrane potential of both oligodendrocytes and neurons, which does not occur in the brain. The stimuli parameters, such as duration, current input, and frequency, need to be optimized for each disease condition. Therefore, it is crucial to carefully evaluate the advantages and disadvantages of TES and TMS using well-established models. In this review, we introduce *in vitro* models employing the compartmentalized microfluidic platform. Despite their unique advantages, these models also have limitations such as gliosis following electrical stimulation and requirement of a transient transfection prior to optogenetic stimulation (Williams et al., 1999; Zhong and Bellamkonda, 2008). Regarding this, there is an urgent need for the development of new tools and models that will become useful in investigating activity-dependent myelination. With a clearer understanding of molecular mechanisms, the modulation of neural activity has the potential to become as a novel therapeutic strategy for treating demyelinating diseases.

## Author Contributions

All authors listed have made a substantial, direct and intellectual contribution to the work, and approved it for publication.

## Conflict of Interest Statement

The authors declare that the research was conducted in the absence of any commercial or financial relationships that could be construed as a potential conflict of interest.

## Abbreviations

BDNF: brain-derived neurotrophic factor
CNS: central nervous system
DRG: dorsal root ganglion
LIF: leukemia inhibitory factor
OPC: oligodendrocyte progenitor cell
PNS: peripheral nervous system
TES: transcranial electrical stimulation
TMS: transcranial magnetic stimulation
TTX: tetrodotoxin.
